# Development and Validation of Predictive Models of Cardiac Mortality and Transplantation in Resynchronization Therapy

**DOI:** 10.5935/abc.20150093

**Published:** 2015-10

**Authors:** Eduardo Arrais Rocha, Francisca Tatiana Moreira Pereira, José Sebastião Abreu, José Wellington O. Lima, Marcelo de Paula Martins Monteiro, Almino Cavalcante Rocha Neto, Camilla Viana Arrais Goés, Ana Gardênia P. Farias, Carlos Roberto Martins Rodrigues Sobrinho, Ana Rosa Pinto Quidute, Maurício Ibrahim Scanavacca

**Affiliations:** 1 Instituto do Coração (InCor) - Universidade de São Paulo, São Paulo, SP - Brazil; 2 Hospital Universitário - Universidade Federal do Ceará, CE - Brazil; 3 Departamento de Saúde Pública - Universidade Estadual do Ceará, Fortaleza CE - Brazil

**Keywords:** Heart Transplantation / mortality, Heart Failure / physiopathology, Cardiac Resynchronization Therapy, Follow-Up Studies, Pacemaker, Artificial

## Abstract

**Background:**

30-40% of cardiac resynchronization therapy cases do not achieve favorable outcomes.

**Objective:**

This study aimed to develop predictive models for the combined endpoint of cardiac death and transplantation (Tx) at different stages of cardiac resynchronization therapy (CRT).

**Methods:**

Prospective observational study of 116 patients aged 64.8 ± 11.1 years, 68.1% of whom had functional class (FC) III and 31.9% had ambulatory class IV. Clinical, electrocardiographic and echocardiographic variables were assessed by using Cox regression and Kaplan-Meier curves.

**Results:**

The cardiac mortality/Tx rate was 16.3% during the follow-up period of 34.0 ± 17.9 months. Prior to implantation, right ventricular dysfunction (RVD), ejection fraction < 25% and use of high doses of diuretics (HDD) increased the risk of cardiac death and Tx by 3.9-, 4.8-, and 5.9-fold, respectively. In the first year after CRT, RVD, HDD and hospitalization due to congestive heart failure increased the risk of death at hazard ratios of 3.5, 5.3, and 12.5, respectively. In the second year after CRT, RVD and FC III/IV were significant risk factors of mortality in the multivariate Cox model. The accuracy rates of the models were 84.6% at preimplantation, 93% in the first year after CRT, and 90.5% in the second year after CRT. The models were validated by bootstrapping.

**Conclusion:**

We developed predictive models of cardiac death and Tx at different stages of CRT based on the analysis of simple and easily obtainable clinical and echocardiographic variables. The models showed good accuracy and adjustment, were validated internally, and are useful in the selection, monitoring and counseling of patients indicated for CRT.

## Introduction

The main international guidelines strongly recommend (class I) cardiac resynchronization therapy (CRT) for patients with congestive heart failure (CHF) and New York Heart Association (NYHA) functional class (FC) II or III or ambulatory class IV when they have intraventricular conduction disturbances and ejection fraction (EF) ≤ 35% while undergoing optimal medical therapy^1^.

However, 30%-40% of CRT cases do not achieve favorable outcomes, which means that these patients undergo surgery with high risks and costs but with no clinical, hemodynamic, or survival benefits^2^. Thus, multifactorial indexes or scores need to be developed to more accurately identify survival predictors and treatment responders^3,4^. Such indexes should involve variables related to mortality reduction, with high rates of sensitivity and specificity.

This work aimed to develop predictive models for the combined endpoint of cardiac death and transplantation (Tx) at different stages of CRT.

## Methods

This prospective observational study evaluated 116 patients with multisite pacemakers implanted consecutively in a tertiary university hospital between January 2008 and March 2013 (Table 1), who had NYHA FC III or ambulatory FC IV (ambulatory outpatients who were taking oral medications), EF ≤ 35%, QRS ≥ 120 ms (left bundle branch block [LBBB] and right bundle branch block [RBBB] with divisional block or pacemaker rhythm), and optimized treatment. The exclusion criteria were severe comorbidities, previous indication for pacemaker implantation, hospitalization for NYHA FC IV heart failure, primary valvular disease, and incomplete data.

**Table 1:** Baseline characteristics and comparison of the results of some variables during the assessment periods

**Variables**	**Time 1**	**Time 2**	**P Value**	**Time 3**	p Value*
Patients	116	114	-	92	-
Age (years)	64.8 ± 11.1	-	-	-	-
Sex (male)	69.8%	-	-	-	-
BMI	25.8 ± 4.1	-	-	-	-
Beta-blockers	88.7%	89.2%	-	91.8%	
ACE-inhibitors	97.4%	96%		95.9%	
Furosemide ≥ 80mg/day	31.9%	17%	-	-	-
Dilated cardiomyopathy	59.4%	-	-	-	-
Ischemic cardiomyopathy	29.3%	-	-	-	-
Chagas disease	11.2%	-	-	-	-
Atrial fibrillation	12%	-	-	-	-
CRT-D	54.2%	-	-	-	-
LBBB	71.55%	-	-	-	-
RBBB with divisional block	12%				
Pacemaker	16.3%				
Posterolateral vein	45.4%				
Anterolateral veins	52.5%				
Prior QRS width (ms)	160	140	< 0.001	-	-
Number of hospitalizations due to CHF	108	24	< 0.001	16	0.79*
Ejection fraction (median)	29%	33%	< 0.001	35%	0.03*
LVDD (mm)	70	66	< 0.001	65	0.73*
Systolic BP (mm Hg)	115	119.6	< 0.001	121.8	0.84*
Diastolic BP (mm Hg)	70	80	0.07	70	0.34*
FC IIl (NYHA)	68.1%	8.7%	< 0.001	12%	0.07*
FC IV (NYHA)	31.9%	6.1%	< 0.001	7.6%	0.07*
DD			< 0.001	-	0.06*
DD Grade I	34.6%	59.2%	-	63.2%	-
DD Grade II	23.7%	27.1%	-	13.9%	-
DD Grade III	29.7%	8.7%	-	16.4%	-
DD Grade IV	11.8%	4.8%	-	5.0%	-
MR	-	-	0.008	-	0.009*
No MR	3.4%	5.3%	-	15.3%	
Mild MR	50.4%	66.0%	-	56.0%	-
Moderate MR	30.4%	18.7%	-	18.6%	-
Severe MR	15.6%	9.8%	-	9.8%	-
RV dysfunction	20.9%	17%	0.62	12%	0.5*
Creatinine (mg/dL)	1.1	1.1	-	1.2	-

Time 1, preimplantation; time 2, 1 year; time 3, 2 years.

* Analysis of time 3 in relation to time 2; QRS width, ejection fraction, left ventricular diastolic diameter and blood pressure were variables without normal distribution (median values); BMI: body mass index; ACE: angiotensin-converting enzyme; CRT-D: cardioverter-defibrillator with biventricular pacing; LBBB: left bundle branch block; RBBB: right bundle branch block; CHF: congestive heart failure; LVDD: left ventricular diastolic diameter; BP: blood pressure; FC: functional class (NYHA); DD: diastolic dysfunction; MR: mitral regurgitation; RV: right ventricle.

Of the 147 patients who underwent implantation during the study period, only 116 were included in the study for the following reasons: 4 had an EF >35%, 3 had total atrioventricular block, 2 had primary valvular heart disease, 2 had pacemaker infection, 7 had incomplete data, 4 had loss of capture in the left ventricle electrode, 2 did not undergo complete follow-up, 1 had severe comorbidity, 5 were hospitalized for class IV CHF at the time of inclusion, and 1 died of premature respiratory infection.

The electrodes of the right ventricle were positioned preferentially in the apical region (84%). The models used in 92, 12, 10, and 2 patients were from St. Jude Medical, Biotronik, Medtronic, and Guidant, respectively. Patients with concomitant indication for an implantable cardioverter-defibrillator (CRT-D group) (54% of the 116 patients) were also included in this study. This indication was for primary prevention in 47 patients and for secondary prevention in 16 patients.

Assessments were performed in the preimplantation period (first analysis), at 1 year after implantation (second analysis), and at 2 years after implantation (third analysis) according to a fixed protocol. We analyzed 12 clinical, 8 electrocardiographic, and 7 echocardiographic variables. The clinical variables were age, sex, body mass index, cardiac cachexia, FC, etiology of cardiomyopathy, cardiac vein where the electrode was positioned in the left ventricle, serum creatinine level, systolic and diastolic blood pressures, use of high-dose loop diuretics (≥ 80 mg/day of furosemide), and hospitalization due to heart failure. The electrocardiographic variables were: atrial fibrillation; LBBB or RBBB; previous cardiac pacemaker; 1^st^-degree atrioventricular block; QRS duration; QRS narrowing after implantation; R wave in the V1 lead in patients with LBBB; and QRS axis in the frontal plane after implantation. The echocardiographic variables were: left ventricular (LV) diastolic and systolic diameters; EF computed using Simpson’s method; degree of diastolic dysfunction (DD) from I to IV; degree of mitral regurgitation from I to III; right ventricular dysfunction (RVD); and dyssynchrony.

A 12-lead surface electrocardiogram was recorded at the speed of 25 mm/s and amplitude of 10 mm/mV. The longest duration of the QRS measured in one of the leads of the frontal or horizontal plane, which was the lead with the highest value and thus allowed for better evaluation, was taken into account. Cardiac mortality was defined for deaths of end-stage CHF or for sudden death.

### Echocardiographic parameters

The echocardiographic guidelines for the analysis of various echocardiographic parameters were followed, as well as the guidelines for dyssynchrony for the analysis of such parameters^5,6^. Three experienced physicians performed the echocardiographic examinations, 72% of which were performed by the same specialist. The examinations were performed using the GE Vivid 7 Ultrasound System (GE Healthcare, Fairfield, CT, USA).

The systolic function analysis of the cardiac chambers was performed using Simpson’s method in the two-dimensional mode. Ventricular diameters were obtained on M-mode echocardiography, according to the standard guideline^5^. Right ventricular function was analyzed qualitatively, differentiated between the presence and absence of any degree of dysfunction^5^.

Diastolic dysfunction analysis was conducted by assessing mitral flow (at rest and after a Valsalva maneuver), tissue Doppler images, and flow propagation speed on color M-mode. Results were used to classify DD into four grades (0, absent; I, mild; II, moderate; III, accentuated or with restrictive dysfunction; and IV, severe or with irreversible restrictive dysfunction)^7^.

The degree of mitral regurgitation was assessed as the percentage of the left atrium filling using color Doppler echocardiography. The percentage was less than 20% in mild reflux, and between 20% and 40% in moderate reflux; values above these percentages indicated severe reflux^5^. In this practical context, the Coanda effect was interpreted as a moderate reflux when restricted to the atrial sidewall and accentuated when it stretched through the upper pole of the left atrium.

All patients provided informed consent, and the ethics committee of the hospital approved the study, whose protocol conforms to the ethical guidelines of the declaration of Helsinki.

### Statistical analysis

The categorical variables were presented as frequencies and percentages, whereas the continuous variables were presented as means and standard deviations, or medians. The categorical variables were compared using the McNemar, Stuart-Maxwell, or chi-square test. The Student *t* test was used to compare the distribution of approximately normal, continuous variables, and the Wilcoxon/Mann-Whitney *U* test was used for the comparisons of continuous variables without normal distribution. Distributions were considered significantly different if p < 0.05.

The univariate relationship between the clinical, electrocardiographic, and echocardiographic variables and the combined endpoint of cardiac mortality and Tx was evaluated by using the Kaplan-Meier survival curve, log-rank test, and Cox regression analysis. Some continuous variables were assessed to determine a cutoff value.

Cox multiple regression models were developed in the following analysis times to assess the independent contribution of each of the significant variables in the Cox univariate model: preimplantation (time 1), first year after CRT (time 2), and second year after CRT (time 3). Variables with p < 10% were considered potential confounders. Each of the variables was included in the multivariate model according to hazard descending order and was excluded when p ≥ 5%. After obtaining the final model, the previously excluded variables were included again in the model and tested individually using the same criteria.

We conducted logistic regression analyses by using hazard^8^ as an independent variable to measure risk, and cardiac death/Tx as the dependent variable. The accuracy of the models was tested with the receiver-operating characteristic (ROC) curve, along with its sensitivity and specificity. Models were prepared by dividing the hazard scores into risk categories according to the number of variables present and classified as low (class A), medium (class B), and high risk (class C).

Kaplan-Meier survival curves were elaborated individually for the independent variables and risk classes, and compared using the log-rank test.

For the proposed models, all the variables were tested for compliance with the proportional hazards assumptions by performing the Schoenfeld test and a visual analysis of the Schoenfeld residuals against the time of deaths or censorship. For each model, the effect of each observation on the estimated parameters was analyzed. To achieve this, after the deletion of an observation, the model was estimated again and the new estimates were compared to the previous ones. Values should not change much or the model may be too sensitive to a particular observation.

To obtain the bootstrap confidence intervals, the original data were sampled 10,000 times to obtain 10,000 pseudo-samples of size 60. Then, for each pseudo-sample, the hazard ratios of the three models were estimated. These estimated hazard rates were sorted, and the 95% confidence interval was reported.

The data were analyzed by using Stata/SE version 12.1 (StataCorp LP, College Station, TX, USA) and the "R" software (2014 -"R": A language and environment for statistical computing. R Foundation for Statistical Computing, Vienna, Austria).

## Results

During the study, 29 deaths were recorded, representing a total mortality rate of 25% during the follow-up period of 34.09 ± 17.9 months. Cardiac mortality/Tx accounted for 16.3% (19 patients) of the cases. Six patients underwent Tx during the study period, 5 for refractory CHF and 1 for recurrent arrhythmic storm. Three Tx patients died prematurely due to disease severity at the time of Tx. No sudden death occurred in the CRT-D group, but sudden death occurred in 3 patients in the CRT-P (pacemaker without defibrillator) group. In the CRT-D group, 6 patients with fast ventricular tachycardia or fibrillation were treated with effective shock. The baseline characteristics of the patients and the comparison of the results of the variables during the assessment period are shown in Table 1.

No significant statistical evidence showed that the assumption of proportional hazards was violated. The effect of each observation on the estimated parameters for each model was analyzed. The data obtained do not suggest influential observations. Bootstrapping confidence intervals for a 95% level of significance were obtained and confirmed the statistical significance of the estimated hazard ratios. These results did not reject the adjustment of the model with the proposed variables (Table 2).

**Table 2:** Bootstrap 95% confidence intervals and formal test for the proportional hazards assumption

**Model 1**			
**Covariate**			**CI**
EF			(1.7142; 14.2053)
RVD			(1.8754; 16.6939)
HDD			(2.2563; 18.7021)
**Model 2**			
CHF			(2.1747; 11.4814)
RVD			(3.0642; 10.6684)
HDD			(4.0963; 18.3712)
**Model 3**			
FC			(3.5177; 37.5661)
RVD			(6.0592; 46.8405)
**Model 1**			
**Covariate**	**ρ**	χ^2^	**p value**
EF	0,073	0,08099	0,776
RVD	0,124	0,28512	0,593
HDD	-0,012	0,00259	0,939
Global		0,33714	0,953
**Model 2**			
ICC	0,3713	1,785	0,182
RVD	0,1089	0,223	0,637
HDD	-0,0934	0,167	0,683
Global		1,905	0,592
**Model 3**			
FC	-0,110	0,118	0,732
RVD	0,125	0,162	0,687
Global		0,254	0,881

CI: confidence interval; EF: ejection traction; RVD: right ventricular dysfunction; CHF: hospitalization due to congestive heart failure; HDD: high doses of diuretic (furosemide ≥ 80 mg/day); FC: functional class (NYHA) III/IV compared with I/II.

### Analysis of the variables at time 1 (preimplantation)

Of the 27 variables analyzed during the first study period (preimplantation), 13 were significant in the Cox univariate regression model. In the Cox multivariate model, RVD, EF <25%, and use of high-dose diuretics (HDD) were independently associated with increased cardiac mortality/Tx, with hazard ratios of 3.9, 4.8, and 5.9, respectively (Table 3).

**Table 3:** Analysis by the Cox model with respect to cardiac mortallty/Tx at time 1 (preimplantation)

**Variable**	**HR**	**95% CI**	**p**	**HR**	**95% CI**	**p**
	**Univariate**			**Multivariate**		
Hospitalization ≥ 1	9.23	1.23-69.21	0.031			
RV dysfunction	5.01	1.97-12.76	0.001	3.95	1.45-10.74	0.007
FC III / IV	4.87	1.85-12.83	0.001			
Chagas Disease	4.73	1.77-12.63	0.002			
EF < 25 %	4.43	1.77-11.05	0.001	4.85	1.71-13.73	0.003
Diuretic ↑	3.89	1.56-9.72	0.004	5.97	2.15-16.53	0.001
SBP < 100 mmHg	3.38	1.35-8.46	0.009			
Creatinine > 1.1	2.85	1.06-7.67	0.038			
LVDD > 80 mm	2.68	1.00-7.15	0.048			
DBP < 60 mmHg	2.63	1.02-6.75	0.044			
ACE inhibitors	4.34	0.98-19.17	0.052			
MR grade II	2.50	0.89-7.41	0.08			
MR grade III	2.80	0.87-9.43	0.08			

HR: hazard ratio (hazard ratio in the Cox model); CI: confidence interval, P: level of statistical significance; Diuretic ↑ ≥ 80mg of furosemide; SBP: systolic blood pressure; DBP: diastolic blood pressure; FCIII / IV: percentage of functional class (FC) III over FCIV; Hospitalization > 1: one or more hospitalizations due to congestive heart failure (CHF); RV: right ventricular; EF: ejection fraction; LVDD: left ventricular diastolic diameter; ACE: angiotensin-converting enzyme; MR: mitral regurgitation.

**Figure 1:**
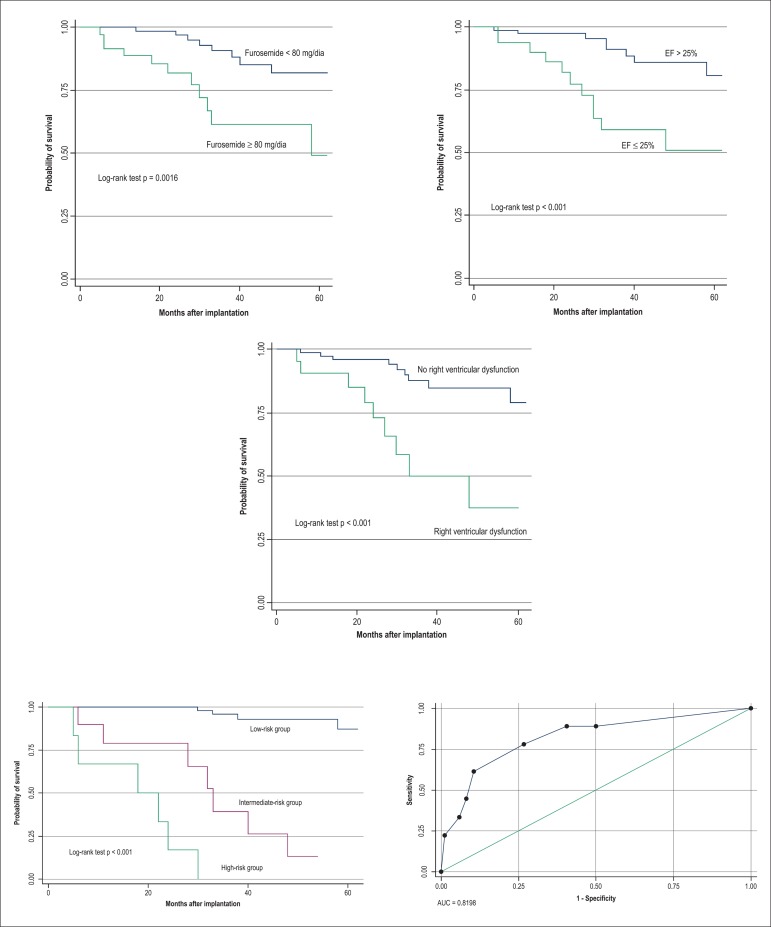
Kaplan-Meier survival curve of the variables with independent value in the multivariate Cox analysis, compared by using the log-rank test, with the construction of the ROC curve, an area under the curve (AUC) of 0.81, sensitivity of 61%, specificity of 89%, and accuracy of 84%. At the bottom right, risk model, being a low risk of cardiac mortality/Tx the absence of the three variables, furosemide> 80 mg/day, right ventricular dysfunction, and ejection fraction (EF) < 25% or presence of one of them.

Significant variables in the multivariate model were also significant in the Kaplan-Meier model when compared using the log-rank test. The analysis of the model by using the ROC curve showed an area under the curve (AUC) of 0.81, with a sensitivity of 61.1%, a specificity of 89.5%, and an accuracy of 84.6% (Figure 1).

From the combinations of these variables, we developed a model with three classes as follows: class A (low risk for cardiac death/Tx) was the absence of the variables or the presence of only one of the significant variables in the multivariate analysis, implying a 30-month cardiac event-free rate of 93%. The combination of two (class B) and three variables (class C) resulted in 30-month cardiac event-free rates of 61% and 0%, respectively.

### Analysis of the variables at time 2 (first year after CRT)

During time 2 (first year after CTR), 13 variables were significant in the Cox univariate regression model. In the Cox multivariate model, RVD, use of HDD, and hospitalization due to CHF were independently related to increased cardiac mortality/Tx rate, with hazard ratios of 3.5, 5.3, and 12.5, respectively.

**Figure 2:**
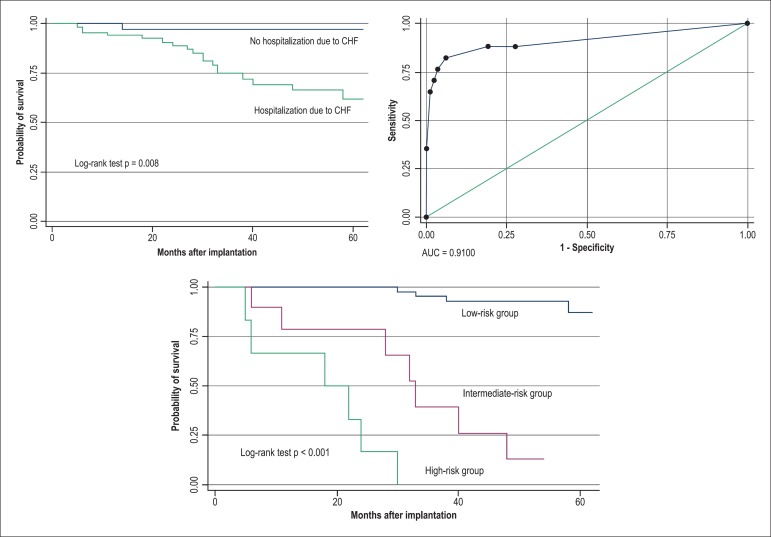
Kaplan-Meier curve of the variable hospitalization due to congestive heart failure (CHF), which, associated with right ventricular dysfunction and use of high doses of diuretics, formed the predictive model of cardiac death/Tx at time 2 (1^st^ year). The absence of the three variables or the presence of only one (low risk) indicates an event‑free rate in 30 months of 98%. At the top right, the ROC curve with an area under the curve (AUC) of 0.91, sensitivity of 76.4%, specificity of 96.3%, and accuracy of 93%.

The significant variables in the multivariate model were also significant in the Kaplan-Meier model, when compared by using the log-rank test. The analysis of the model by using the ROC curve showed an AUC of 0.910, with a sensitivity of 76.4%, a specificity of 96.3%, and an accuracy of 93% (Figure 2).

From the combinations of these variables, we were able to construct a model with three classes (Table 4). Class A means low risk of cardiac death/Tx, composed by the absence or presence of only one of the significant variables in the multivariate analysis, resulting in a 30-month cardiac event-free rate of 98%. The combination of two (class B) and three variables (class C) resulted in 30-month cardiac event-free rates of 65% and 0%, respectively (Figure 2).

**Table 4:** Predictive scores of cardiac mortality and transplantation In cardiac resynchronlzatlon therapy Score at time 1 (preimplantation)

**Variable**	**Hazard**	**N**	**Scores**	**Class**	**Risk**
None	1.0	45	0	A,	Low
RVD	3.9	8	3		Low
EF	4.8	14	4		Low
Diuretic ↑	5.9	17	5		Low
RVD + EF	19.1	5	7	B	Intermediate
RVD + Diuretic ↑	23.6	4	8	B	Intermediate
EF + Diuretic ↑	29.0	6	9	B	Intermediate
RVD + EF + Diuretic ↑	114.0	5	12	C	High
					
**Score at time 2 (1^st^ year)**					
**Variable**	**Hazard**	**N**	**Scores**	**Class**	**Risk**
None	1.0	62	0	A	Low
RVD	3.5	7	2	A	Low
Diuretic ↑	5.3	12	3	A	Low
Hospitalization	12.5	3	5	A	Low
RVD + Diuretic ↑	18.7	2	6*	B	Intermediate
RVD + Hospitalization	44.0	2	7	B	Intermediate
Diuretic ↑ + Hospitalization	66.3	6	8	B	Intermediate
RVD + Hospitalization + Diuretic ↑	234.0	6	10	C	High
					
**Score at time 3 (2^nd^ year)**					
**Variable**	**Hazard**	**N**	**Scores**	**Class**	**Risk**
None	1.0	55	0	A	Low
FC III/IV	7.7	10	8	B	Intermediate
RVD	12.1	4	13	B	Intermediate
RVD + FC III/IV	94.5	5	21	C	High

RVD: right ventricular dysfunction; EF: ejection fraction lower than 25%; diuretic ↑: use of ≥ 80 mg of furosemide; FC: functional class (NYHA); Hospitalization: ≥ 1 hospitalization due to congestive heart failure. Class A: low risk category Class B: intermediate risk and Class C: high risk.

The hazard was used as an independent variable in the logistic regression model for the preparation of the score. The score was obtained by the hazard ratio of the variable divided by the highest value.

* one unit was added to maintain the hazard proportion. N: number of patients.

### Analysis of the variables at time 3 (second year after CRT)

Hospitalizations due to CHF, use of HDD, FC, DD, RVD, EF < 30%, Chagas disease, and systolic blood pressure < 110 mmHg were significant in the univariate Cox regression model in the second year after CRT.

**Figure 3:**
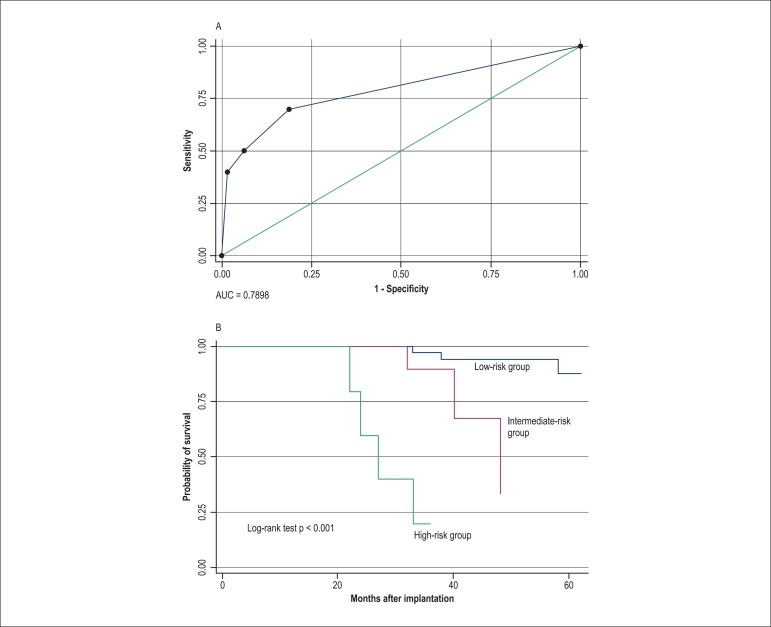
A: ROC curve of the model at time 3 (2nd year), with the variables right ventricle (RV) dysfunction and functional class (FC) III and IV compared with I and II, with an area under the curve (AUC) of 0.789, sensitivity of 40%, specificity of 98.4% and accuracy of 90.5%. B: Kaplan-Meier curve showing that the absence of the variables RV dysfunction and FC III / IV (Class A - low risk) indicates an event-free rate of 97.5% at 30 months.

In the multivariate Cox model, RVD and FC III/IV were independently related to increased cardiac mortality/Tx rate, with hazard ratios of 7.7 and 12.0, respectively. The significant variables in the multivariate model were also separately significant in the Kaplan-Meier model when compared using the log-rank test (p < 0.001). The analysis of the model using the ROC curve showed an AUC of 0.789, with a sensitivity of 40%, a specificity of 98.4%, and an accuracy of 90.5% (Figure 3).

From the combination of these variables, we were able to construct a model with three classes. Class A means low risk of cardiac death/Tx, composed by the absence of the two significant variables in the multivariate analysis, implying a 30-month cardiac event-free rate of 97.5%. The presence of the combination of two (class B) and three variables (class C) resulted in 30-month cardiac event-free rates of 83.1% and 38.5%, respectively.

## Discussion

In the present study, we developed three predictive models for the risk of cardiac death and Tx at different stages of CRT. To our knowledge, this is the first study to sequentially and prospectively analyze predictive variables in the same population and at different stages of development (at preimplantation, in the first year after CRT, and in the second year after CRT) and to develop risk models for cardiac death/Tx. The models identified simple variables that, when present, were associated with a high risk for cardiac death/Tx.

The total mortality rate was 25% (29/116) at 34 ± 17 months. In the CARE-HF study^9^, the mortality was 30% in the group without intervention, compared with 20% in the group with CRT, during a 29.4-month follow-up. In the COMPANION study^10^, the mortality rate was 21% (131/617) in the CRT group, compared with 25% (77/308) in the control group, during a 24-month follow-up. Therefore, our total mortality data are within the range described by large-scale studies. In our study, we analyzed the combined endpoints of cardiac mortality and Tx, aiming at identifying more-specific variables related to CRT results^11^.

Several studies have evaluated predictors of response or death in different populations and with different response criteria, and the results were inconsistent. However, several publications identified the following predictors of response: dilated cardiomyopathy^12^, QRS width^13^, QRS narrowing^14^, presence of dyssynchrony^15^, female sex^16^, type of bundle branch block^17^, LV diameter^18^, the aortic velocity time integral^13^, and DD^19^.

The patients with RVD (20.9% of the group) had worse evolution in all the analysis times. However, we noticed that 6 patients with good outcomes had regression of the alterations in the right ventricle. The study by Praus et al^20^ showed that the regression of the right ventricle occurred later (15 months), whereas Leong et al^21^ identified the right ventricle as an independent predictor of mortality. Therefore, patients with RVD should not be excluded from the indication for CRT, although they represent a subgroup at higher risk of cardiac death or Tx after CRT^22^. The importance of the right ventricle in CRT has been demonstrated in other recent studies, but not in the elaboration of risk models for different evolution stages^23,24^.

Thirteen patients (11.2%) had Chagas disease, 5 of whom had RVD. Chagas cardiomyopathy was related to increased mortality in the survival curve, similar to another study that related it with worse outcome^25^. In the multivariate analysis, Chagas disease did not remain as an independent variable, probably because 41% of the patients had RVD, a variable that was significant at all the analysis times. Therefore, the relevance of RVD was not exclusively related to Chagas disease, as 19 patients had RVD due to other etiologies.

A preimplantation EF <25% identified a subgroup with the highest risk for cardiac death. Linde et al^26^, in a subanalysis of the REVERSE study, have shown that a basal EF <30%, compared with values between 30% and 40%, was positively related to survival. Meanwhile, Kronborg et al^27^ showed that a basal EF < 22.5% determined an increased mortality after CRT.

The hospitalizations for heart failure proved to be an independent variable in relation to the prediction of cardiac mortality/Tx in the first year after CRT. The study represents, to our knowledge, the first time this variable was included as independent in the analysis of mortality risk in the first year after CRT and not as part of the outcome combined with death. Hospitalization due to CHF is a well-defined risk factor for cardiomyopathy, with a reduction in the incidence of these events after CRT demonstrated in several studies^9,10^.

Another easily obtainable clinical variable that showed significant value in the preimplantation period and first year after CRT was the use of high-dose loop diuretics (furosemide ≥ 80 mg/day). Van Boven et al^28^ reported an association between chronic non-use of diuretics and response to CRT. Meanwhile, Cleland et al^29^ observed that the use of HDD was related to a worse prognosis only in the univariate analysis. We believe that the description of this variable as an independent value of cardiac death in two periods of the CRT analysis in our study is an original observation.

A clinical prediction rule to identify patients at heightened risk for early demise after CRT has been recently elaborated^30^, including the following four independent variables: LV end-diastolic diameter (LVEDD) > 65 mm, non-LBBB morphology, creatinine level > 1.5, and non-use of beta-blockers. In our study, LVEDD and creatinine level were significant only in the univariate analyses. Hospitalization due to CHF, use of HDD, and RVD, some independent variables in our work, were not included in the previous study.

We achieved a significant improvement in the specificity of the predictive models of mortality or response after CRT, reaching 96% in the first year after CTR and 98% in the second year after CRT, when compared with the specificity of 22%-70% of previously described models in relation to total and cardiac mortality. These results are in accordance with the target outcomes of CRT in the treatment of patients with severe illnesses, with high costs and risks in the procedure^31^.The models used in this study showed good accuracy, ranging from 84.6% to 93%, and can be used in three different stages of CRT, which is another original contribution of our work. At the usual significance levels, the model was validated internally and did not reveal lack of adjustment or exaggerated sensitivity to the data.

We believe that the study contributes to and advances the search for better criteria for prognostic evaluation, with the composition of simple multifactorial indexes and with the inclusion of easily obtainable variables that are used in clinical practice. The models will be useful in the selection, monitoring, and counseling of patients indicated for CRT.

### Study limitations

Analyses of intraobserver and interobserver variabilities of echocardiographic and electrocardiographic parameters were not performed. The patients did not undergo optimization of the atrioventricular interval after surgery. The models created were not validated externally, although they were validated internally. This study is also limited by the small number of patients, the large number of excluded patients, and the fact that it was conducted at a single center. The RV function was analyzed qualitatively due to the absence of correlation between the RV measures and the prognosis at the beginning of the study. These results must be considered within the study population, who had 59.4% of dilated cardiomyopathy, 11.2% of Chagas cardiomyopathy, 12% of patients with RBBB and 16.3% of patients with prior cardiac pacemaker. Future larger prospective studies will help validate the important variables related to cardiac death or Tx after CRT.

## Conclusion

We developed predictive models of cardiac death or Tx at different stages of CRT based on the analysis of simple and easily obtainable clinical and echocardiographic variables. The models showed good accuracy and adjustment, were validated internally, and are useful in the selection, monitoring, and counseling of patients indicated for CRT.
